# Case Report: Diagnosis of Mucopolysaccharidosis Type IVA With Compound Heterozygous Galactosamine-6 Sulfatase Variants and Biopsy of Replaced Femoral Heads

**DOI:** 10.3389/fped.2022.914889

**Published:** 2022-07-04

**Authors:** Yiyang Ma, Hao Peng, Fuchou Hsiang, Haoyu Fang, Dajiang Du, Chenyi Jiang, Yehui Wang, Chun Chen, Changqing Zhang, Yun Gao

**Affiliations:** Department of Orthopedic Surgery, Shanghai Sixth People’s Hospital, Shanghai, China

**Keywords:** Morquio A Syndrome, femoral head, micro-CT, GALNS, WES, compound heterozygous variants

## Abstract

**Introduction:**

Mucopolysaccharidosis Type IVA (MPS IVA) or Morquio A Syndrome, is a rare metabolic disorder caused by compromised galactosamine-6 sulfatase (GALNS) encoded by *GALNS* gene (NM_000512.5), leading to keratin sulfate (KS), and chondroitin-6-sulfate accumulation in various organs. We present a 17-year-old woman with progressive bilateral hip pain and radiographic evidence of spondyloepiphyseal dysplasia.

**Methods:**

Diagnosis of MPS IVA was made based on whole-exome sequencing (WES) of blood samples collected from the patient and family members, high urinary glycosaminoglycan excretion, supportive clinical manifestations, radiographic examinations, including whole-body X-rays, cervical MRI, and pelvic CT. The patient underwent bilateral total hip arthroplasties sequentially, at a 1-month interval. Femoral heads were preserved for the micro-CT (μCT) analysis and the osteochondral histology examination.

**Results:**

The patient presented with multiple skeletal deformities, including vertebras and long bone deformities. WES disclosed compound heterozygous variants at exon 11 (c.1156C>T) and exon 12 (c.1288C>G) of the *GALNS* (NM_000512.5). The μCT analysis revealed significant bone quantity loss and microarchitectural change in both weight-bearing area (WBA) and non-weight-bearing area (NWBA) of the femoral heads, while histological analysis showed structural abnormity of articular cartilage in the WBA of the femoral heads.

**Conclusion:**

We have found compound heterozygous variants of *GALNS*. This is also the first study to report the microarchitectural and histological changes of both subchondral bone and articular cartilage of the femoral head in a patient with MPS IVA.

## Introduction

Mucopolysaccharidosis Type IVA (MPS IVA), namely Morquio A Syndrome, is a rare autosomal recessive metabolic disorder caused by functionally deficient N-acetylgalactosamine-6-sulfatase encoded by the *GALNS* gene (NM_000512.5), resulting in multi-system accumulation of keratan sulfate (KS) and chondroitin-6-sulfate (1). Glycosaminoglycan (GAG) accumulation impairs epiphyseal development by delaying ossification and eventually leads to skeletal dysplasia, associated quality of life impairment, and in some cases, shortened lifespan (2).

## Case Description

A 17-year-old (y/o) woman presented with progressive pain in her bilateral hips. The patient was a wheelchair user and was previously diagnosed with developmental dysplasia of the hip (DDH), seeking hip replacement surgery. Additionally, she had shallow acetabuli and flattened femoral heads (Figure 2). Radiology noticed spondyloepiphyseal dysplasia, suggestive of a genetic cause. The urinary sample was collected for the GAG level test, and a blood sample was collected for genotyping *via* whole-exome sequencing (WES).

WES discovered compound heterozygous variants of *GALNS* (NM_000512.5). Together with clinical manifestations, radiographic and laboratory tests, MPS IVA was diagnosed (1). The patient received bilateral total hip arthroplasties (THAs) sequentially and regained mobility. The patient is now under supportive therapy elsewhere. Femoral heads were preserved after surgeries. In this case, we will review the epiphyseal parameters *via* micro-CT (μCT) and histology analysis in the MPS IVA femoral heads with the patient/guardian’s consent.

### Subject

**FIGURE 1:**
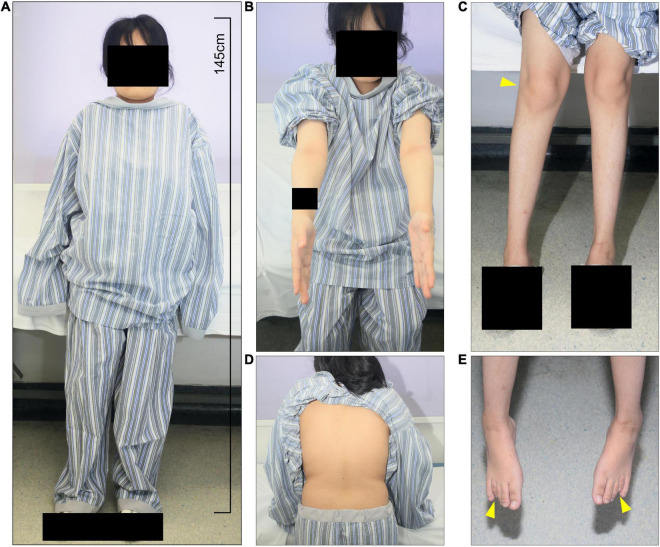
The patient presents short stature and systemic skeletal deformities. **(A)** At admittance, the patient was 145 cm tall with normal intelligence with multiple skeletal deformities, including **(B)** elbow and **(C)** knee and ankle valgus, and **(E)** 4th metatarsal shortening. **(D)** Scoliosis and kyphosis were not present.

**FIGURE 2:**
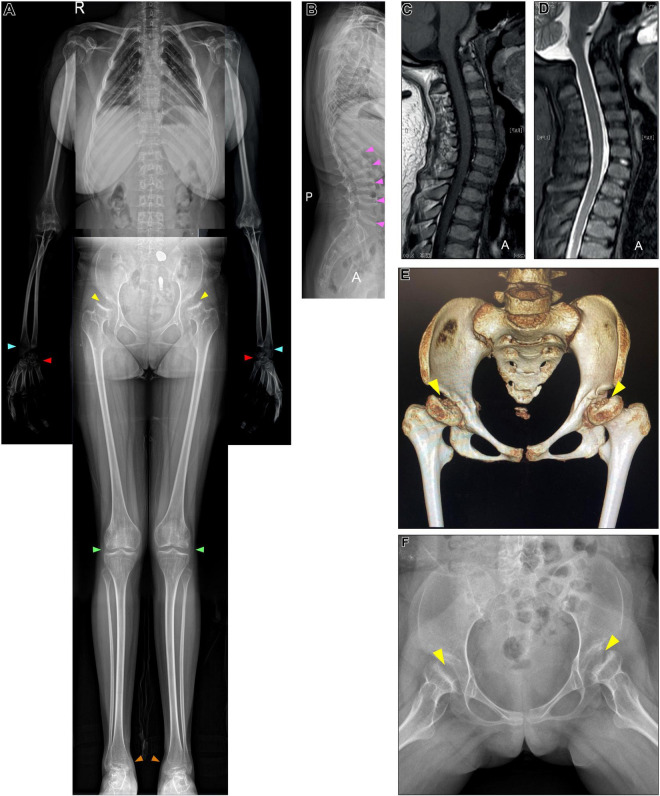
X-ray, CT of the pelvis, and cervical MRI examination. **(A,B)** Whole-body X-ray found multiple skeletal deformities, including vertebral wedging (pink arrows), small irregular carpal bones (red arrows), the distal portion of the radius being tilted toward the ulna (blue arrows), mild genu valgum (green arrows), and ankle valgus (orange arrows). **(A,F)** Pelvis X-ray and **(E)** CT collectively show bilateral acetabular dysplasia, and femoral heads collapse (yellow arrows). **(C,D)** MRI excluded cervical stenosis.

We studied a 17 y/o woman with a complaint of progressive walking pain in bilateral hips which gradually disabled her from any motion of lower extremities since the clinical manifestation debuted at her age of 8. At admittance, the patient was 145 cm tall with normal intelligence. Whole-body skeletal radiographs were obtained and found mild dysostosis multiplex (Figures 1, 2). Extremity deformities involve small irregular carpal bones, the ulnar deviation of the distal portion of the radius, mild genu valgum, ankle valgus, and 4th metatarsal shortening (Figures 1, 2). Cervical stenosis was excluded by MRI (Figures 2C,D).

### Whole Exome Sequencing

**FIGURE 3:**
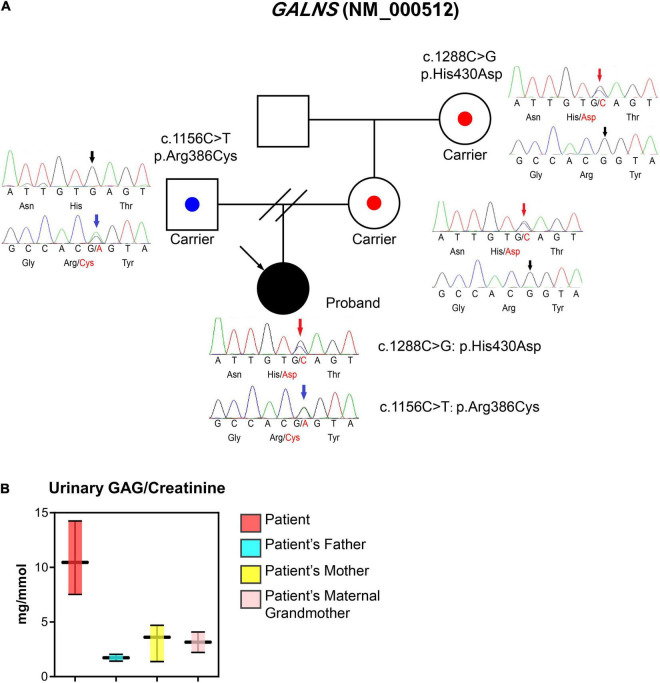
Laboratory tests, the pedigree of the patient, and sequencing information. **(A)** Proband is a 17-year-old woman with a major complaint of progressive walking pain in bilateral hips which gradually disabled her from any motion of lower extremities since the clinical manifestation debuted at her age of 8. We discovered compound heterozygous variants at exon 11 (c.1156C>T; blue arrows) and exon 12 (c.1288C>G; red arrows) of the *GALNS* gene, respectively. The sequencing results indicate both parents are respective carriers and each donated a different heterozygous allele. **(B)** Urinary GAG/Creatinine (mg/mmol) was measured using samples collected from MPS IVA patient and their family members during hospital visits.

WES was performed due to the radiographic findings of systemic skeletal anomalies which suggested a genetic cause. Targeted capture high-throughput WES revealed two heterozygous variants, Exon 11 (NM_000512.5:c.1156C>T) and Exon 12 (NM_000512.5:c.1288C>G) of the *GALNS* gene, respectively, in this patient (Figure 3A).

To confirm the phase of variants, WES was performed on the patient’s parents and maternal grandmother who are free of the clinical phenotype. Parental testing confirmed that variants are in *trans* (Figure 3A). Sanger sequencing confirmed the occurrence of the compound heterozygous *GALNS* variants in the patient.

### Urinary Excretion of Glycosaminoglycan Measurement Using Dimethylmethylene Blue Method

The first morning void urine was collected from the patient, mother, and maternal grandmother. The urinary excretion of GAG (uGAG) was measured by the Dimethylmethylene Blue (DMMB) method and was normalized with urinary creatinine (3). The uGAG content was significantly higher in the patient’s urine when normalized to urine creatinine, while the patient’s family members exhibited normal levels (Figure 3B).

### Micro-CT Analysis of Femoral Heads

**FIGURE 4:**
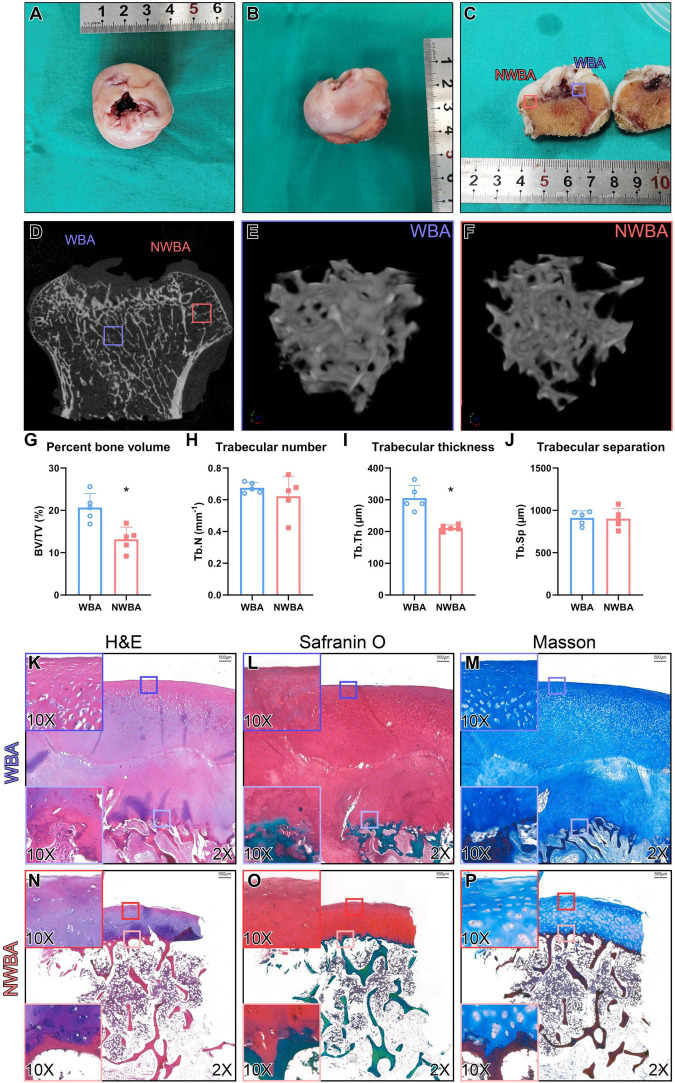
Micro-computed tomography and histology analysis of femoral heads from a 17-year-old woman with MPS IVA (Patient). **(A,B)** Femoral heads were collected from a 17-year-old woman with MPS IVA, showing saddle-shaped and collapsed articular surface. **(C)** Areas of articular cartilage from the weight-bearing area (WBA, blue square) and non-weight-bearing area (NWBA, red square) were collected for histology staining. **(D–F)** Areas of subchondral bone from WBA (blue square) and NWBA (red square) were collected for μCT scans. **(G)** Bone volume fraction (BV/TV,%), **(H)** trabecular number (Tb.N, mm^–1^), **(I)** trabecular thickness (Tb.Th, mm), and **(J)** trabecular separation (Tb.Sp, mm) of subchondral bone from WBA and NWBA were calculated using CTAn software, respectively. **(K–P)** Sections from WBA and NWBA were stained with H&E staining, Safranin O and Masson. “*” represents *P*-value < 0.05.

Bilateral femoral heads were preserved after THAs for μCT analysis (Figures 4A–F). Percentage bone volume (BV/TV, *P* = 0.005) and trabecular thickness (Tb.Th, *P* < 0.001) were significantly lower in the non-weight-bearing area (NWBA) when compared to the weight-bearing area (WBA), while the trabecular number and separation were indistinguishable (Figures 4G–J).

### Histological Evaluation of Femoral Head

Sections of the articular surface from both WBA and NWBA of the preserved femoral heads were stained with hematoxylin & eosin (H&E), Safranin O/Fast green, or Masson to evaluate the morphological change of cartilage and subchondral bone in the femoral heads of MPS IVA patient. In the WBA of the femoral head, the articular cartilage was abnormally thick and divided into two layers (Figures 4K–M). The superficial layer exhibited fibrous change, while the chondrocytes in the middle zone appeared to be hypertrophic. The disorganized chondrocytes were accompanied by clusters formation. In the deeper layer, the subchondral bone was filled with metaplastic tissue composed of irregular fibrous-like structure with no mature chondrocytes seen. Both layers of WBA were strongly stained by Safranin-O, indicating GAG accumulation (Figures 4L,O). In the NWBA, the thickness of the articular chondrocytes appeared near-normal and chondrocytes cluster formation was found throughout the cartilage layer, while the subchondral bone in the NWBA was discontinuous (Figures 4N–P).

## Discussion

Our case reports diagnosing a 17 y/o woman MPS IVA patient and investigating her replaced femoral head *via* histology and μCT for articular cartilage and subchondral bone analysis. The initial symptom in our case was mobility impairment which debuted at the age of 8. The patient reached a final height of 145 cm by 17 y/o as closed growth plates were seen on her whole-body skeletal radiographs. Mild genu valgum, ankle valgus, small irregular carpal bones, and the ulnar deviation of the distal portion of the radius were noted while no pectus carinatus, kyphoscoliosis, or cervical stenosis was found. Corneal clouding and hearing problems were not present in our case while cardiac valvular involvement was not investigated. Therefore, combining her clinical manifestations and radiology findings, her severity falls into the mild tier according to Tüysüz et al. and Montano et al.’s classifications (4). Additionally, the shortness of bilateral 4th metatarsal bones presented in our case is consistent with the observations made from attenuated forms reported by Tüysüz et al. earlier (5).

The incidence of MPS IVA ranges from 1/71,000 to 1/1,872,000 (6). Due to the rareness of MPS IVA prevalence and severity variation, hip pathology in MPS IVA can be confused with skeletal dysplasia, such as DDH, Legg–Calvé–Perthes disease (LCPD), Spondyloepiphyseal dysplasia congenita (SEDC), or other hip pathology (5). Therefore, to make an accurate diagnosis, the systemic investigation is a must. We have successfully diagnosed this case using molecular tests and provided the bone parameters and cartilage histology of the capital femoral epiphyses in an MPS IVA patient.

The DDH is characterized by the congenital inability of acetabulum dysplasia to cover the femoral head which cannot be explained from the genetic aspect (7). While in MPS IVA, the incongruity of the femoral head and acetabulum is led by the capital femoral epiphyses dysplasia. Though DDH and MPS IVA share the structural abnormality of the acetabulum or femoral head with continuous Shenton’s line (8), neonatal DDH screening, systemic investigations, blood or urinary metabolic test, and genotyping can easily distinguish one from another.

Legg–Calvé–Perthes disease causes unilateral or bilateral avascular necrosis of femoral heads in children and has multiple etiologies (9). The necrotic bone gradually collapses, and the femoral head hence loses its sphericity, resulting in a permanent deformity. Due to the resultant joint incongruence, hip pain and stiffness will occur, which usually debut between the ages of 4 to 15. Therefore, LCPD patients present reminiscent hip manifestation and radiographic findings of femoral heads as presented in this patient. However, the local and unilateral onset of LCPD, in association with a complete metabolic panel study, will accurately differentiate LCPD from MPS.

Spondyloepiphyseal dysplasia congenita is an autosomal dominant disorder caused by variants in the *COL2A1* gene. The presentations of SEDC resemble those of MPS IVA in many aspects, such as short stature, short neck, spinal and epiphyseal dysplasia, lower limb deformities, and possible vision and hearing problems (10). Unlike the hypermobile joints in MPS IVA patients, joint mobility decreases in SEDC, which is a unique diagnostic feature to differentiate MPS IVA from SEDC (4).

In this case, we have found two heterozygous missense variants of *GALNS* at exon 11 (NM_000512.5:c.1156C>T) and exon 12 (NM_000512.5:c.1288C>G) using targeted capture high-throughput WES which was verified by Sanger sequencing. Both variants show 0 frequency in the east Asian population according to gnomAD (PM2_Supporting). Missense variant in *GALNS* with a low rate of benign missense mutations and for which missense mutation is a common mechanism of MPS IVA (PP2) while both variants are “deleterious” based on REVEL prediction (NM_000512.5:c.1156C>T = 0.85, NM_000512.5:c.1288C>G = 0.70, PP3; 11). The paternally inherited c.1156C>T variant has been previously reported (PP5; 12, 13). *In vitro* functional experiments indicated that this variant could affect the enzyme activity of GALNS by altering the normal function of the coding protein (PS3; 13, 14). 6 pathogenic or likely pathogenic reported variants were found in a 38bp region surrounding this variant in exon 11 without any missense benign variants (PM1). This variant has been reported in *trans* with the known pathogenic variant c.1364+1G>A (PM3; 15). Thus, the c.1156C>T variant was categorized as “pathogenic” (PS3 + PM1 + PM3 + PM2_Supporting + PP2 + PP3 + PP5) according to the 2015 American College of Medical Genetics and Genomics-Association for Molecular Pathology guidelines (16). The maternal c.1288C>G missense mutation has been recently reported by our colleagues (17). Our studies revealed that this variation is maternally inherited and is in *trans* with c.1156C>T (PM3). Based on the pedigree information, unique clinical findings, and molecular tests (PP4), we can categorize this variation as “likely pathogenic” (PM3 + PM2_Supporting + PP2 + PP3 + PP4; 16).

In MPS IVA patients, due to the KS storage in the cartilage, the mineralization process is hampered, leading to compromised ossification and osteoporosis (18). BMD in the lumbar spine has been reported to be decreased in MPS IVA patients (19), yet lacking further study of epiphyses. Previous understanding of the MPS IVA hip deformity was limited only to radiography. To our knowledge, this is the first report to analyze simultaneously the gross anatomy and microarchitecture of MPS IVA capital femoral epiphyses. In our study, the femoral heads were saddle-shaped with collapsed WBA surface, which is consistent with previous publications (2). The bone volume and trabecular bone thickness in the NWBA were significantly lower than those found in the WBA, indicating subchondral bone deterioration in the NWBA of MPS IVA femoral heads. However, it is currently impossible to comprehend whether the decreased bone quantity was directly caused by KS accumulation or reduced mechanical stimuli due to attenuated mobility caused by hip pain. By comparing μCT results of MPS IVA femoral heads with published microarchitecture parameters obtained from femoral heads of either patient with OA secondary to residual hip dysplasia (HD-OA, *N* = 20, 64.25 ± 5.20 y/o) or patients with osteoporosis (OP, *N* = 20, 67.15 ± 7.7 y/o), MPS IVA femoral heads exhibit subchondral bone inferiority in terms of lower BV/TV and Tb.N, higher Tb.Sp. Only the WBA Tb.Th of MPS IVA falls between those of HD-OA and OP. Thus, we can indirectly apprehend that the overall subchondral bone quantity loss in the MPS IVA femoral heads favors the genetic cause.

Our study is the first histological analysis of the femoral head of a patient with MPS IVA to verify the previous radiographic observation. Instead of previously reported ossified femoral heads (2), we observed thickening of articular cartilage and metaplastic cartilage structure beneath the articular surface in the WBA, profound articular chondrocyte clustering in NWBA than in WBA, and disappearance of chondrocyte columnization at an osteochondral junction in both areas, indicating epiphyseal closure. Chondrocyte clusters are usually found in arthritic articular cartilage, at the site of increased biosynthetic activity (20), representing the chondrocyte replication in response to the adjacent ECM damage and cell death, followed by migration, differentiation, and new matrix formation (21). Therefore, chondrocyte clustering observed in the NWBA might be associated with aberrant matrix metabolisms, such as KS accumulation, and cartilage degeneration (22). Additionally, we speculate the articular disarrangement in the WBA might also be secondary to osteochondral unit instability. However, whether the aforementioned discovery of the metaplastic cartilage layer is compensatory or resultant of the compromised ossification is currently unknown. Collectively, our study has filled the histology gap of capital femoral epiphyses in MPS IVA by providing a comprehensive understanding of the osteochondral pathology in the MPS IVA hip.

To conclude, we have found that the femoral head exhibited collapsed and flattening shape with articular thickening and bone loss in an MPS IVA patient with compound heterozygous variants of *GALNS*. The knowledge we have obtained from this case sheds light upon the pathogenesis of epiphyses dysplasia in the femoral head of MPS IVA patients at the trabecular and histological levels. Early diagnosis and intervention will clinically benefit the patient by improving mobility.

## Data Availability Statement

The datasets presented in this study can be found in online repositories. The names of the repository/repositories and accession number(s) can be found below: https://www.ncbi.nlm.nih.gov/, PRJNA824281.

## Ethics Statement

The studies involving human participants were reviewed and approved by Human Ethics Committee of Shanghai Sixth People’s Hospital, Shanghai Jiao Tong University in China. Written informed consent to participate in this study was provided by the participants’ legal guardian/next of kin. Written informed consent was obtained from the individual(s), and minor(s)’ legal guardian/next of kin, for the publication of any potentially identifiable images or data included in this article.

## Author Contributions

YM: writing – original draft, investigation, and visualization. HP: writing – original draft, investigation, and data curation. FH, HF, CJ, and YW: investigation. DD: investigation and methodology. CC: resources. YG: conceptualization, visualization, supervision, and writing – review and editing. CZ: resources, supervision, conceptualization, and writing – review and editing. All authors contributed to the article and approved the submitted version.

## Conflict of Interest

The authors declare that the research was conducted in the absence of any commercial or financial relationships that could be construed as a potential conflict of interest.

## Publisher’s Note

All claims expressed in this article are solely those of the authors and do not necessarily represent those of their affiliated organizations, or those of the publisher, the editors and the reviewers. Any product that may be evaluated in this article, or claim that may be made by its manufacturer, is not guaranteed or endorsed by the publisher.
