# Supplementary material for: Case Report: Diagnosis of Mucopolysaccharidosis Type IVA With Compound Heterozygous Galactosamine-6 Sulfatase Variants and Biopsy of Replaced Femoral Heads
Source: Front Pediatr. 2022 Jul 4;10:914889. doi: 10.3389/fped.2022.914889 (PMC9289150; doi:10.3389/fped.2022.914889)
Supplement: Supplementary file 1 [file Data_Sheet_1.docx]

**Supplementary Material**

## Methods

### Whole exome sequencing (WES)

WES of MPS patients and family members was performed on DNA extracted from circulating leukocytes. The libraries were constructed using SureSelect Clinical Research Exome kit (Agilent) and sequenced using Illumina HiSeq platform. Clean reads obtained after filtration were aligned to the reference genome (UCSC hg19) using Burrows-Wheeler Aligner (BWA mem). The duplicated reads were removed using Picard tool. SNV (Single Nucleotide Variant) and small InDel (small Insertion and Deletion) were detected using Genome Analysis Toolkit (GATK). Annotation of variants and genes was performed using ANNOVAR. Variant classification was based on criteria provided by the American College of Medical Genetics and Genomics (ACMG).

### Urinary excretion of GAG measurement using dimethylmethylene blue (DMMB) method

To prepare 1L DMMB dye solution, dissolve 16 mg DMMB (Sigma Chemical, St. Louis, MO, USA) in 1L water containing 3.04g glycine (Sigma Chemical, St. Louis, MO, USA), 1.6g NaCl and 95ml of 0.1M Acetic Acid (Yonghua chemical). Pipet 20µl of 10-fold diluted urine sample into the 96 well microplate and add 200µl of DMMB dye solution to each sample and shake the plate of a plate shaker for 5sec. The metachromatic reaction of GAG with DMMB was monitored spectrophotometrically at 525nm using Thermo Varioscan LUX (Singapore, Singapore). The GAG/creatinine ratio (milligrams of GAG per millimole of creatinine) was used to quantify the urinary excretion of GAG.

### Creatinine measurement

Mix 0.2M NaOH and saturated picric acid (Sigma Chemical, St. Louis, MO, USA) in a ratio of 1:1 to prepare dye solution. Pipet 20µl of Creatinine standard (Meilun Biotechnology, Dalian, China) or 10-fold diluted urine sample into the 96 well microplate. Add 200µl of dye solution to each sample and incubate in RT for 20min. The metachromatic reaction was monitored spectrophotometrically at 490nm using Thermo Varioscan LUX (Singapore, Singapore).

### Micro-CT (µCT) analysis of femoral heads

A total of 15 cuboid specimens of trabecular bone were extracted from bilateral femoral heads, of which 5 were located at subchondral region, 5 were located at epiphyseal region and 5 were located at metaphyseal region. After fixed in 4% paraformaldehyde, all samples were scanned with a 9-micron voxel size micro-CT scanner (Skyscan 1176, Kontich, Belgium). The acquisition conditions are 35kV of energy and 220mA of intensity. After scanning, a representative cuboid region with 2.7 mm side length were selected from each specimen. Reconstructed images were managed by CTVox software (v 3.0, Bruker, Belgium). The microarchitectural parameters including bone surface density (BS/TV) (%), bone volume fraction (BV/TV), trabecular thickness (Tb.Th), trabecular separation (Tb.Sp), trabecular number (Tb.N) were calculated using CTAn software (v 1.17, Bruker, Belgium).

### Histological evaluation of femoral head

Specimens of cortical bone and cartilage from both weight bearing area and non-weight bearing area were extracted. Each specimen was fixed in 4% formalin, then decalcified with 10% EDTA and embedded in paraffin. The specimens were sliced in 7 um thickness and stained with hematoxylin & eosin (H&E), Safranin O/Fast green staining or Masson staining.

### Statistical analysis

All data are presented as means ± standard deviations (SD). The differences between groups were determined by Student's t test using Prism 8 software (GraphPad). P<0.05 was considered statistically significant.
